# Clinical practice guidelines and consensus statements for antenatal oral healthcare: An assessment of their methodological quality and content of recommendations

**DOI:** 10.1371/journal.pone.0263444

**Published:** 2022-02-03

**Authors:** Annika Wilson, Ha Hoang, Heather Bridgman, Leonard Crocombe, Silvana Bettiol

**Affiliations:** 1 Centre for Rural Health, College of Health and Medicine, University of Tasmania, Launceston, Tasmania, Australia; 2 Dentistry & Oral Health, Rural Health School, La Trobe University, Bendigo, Victoria, Australia; 3 Tasmanian School of Medicine, College of Health and Medicine, University of Tasmania, Hobart, Tasmania, Australia; School of Dentistry, International Medical University, MALAYSIA

## Abstract

**Objectives:**

To review the content of recommendations within antenatal oral healthcare guidance documents and appraise the quality of their methodology to inform areas of development, clinical practice, and research focus.

**Method:**

A systematic search of five electronic databases, Google search engine, and databases from relevant professional and guideline development groups published in English, developed countries, and between 2010 and 2020 was undertaken to identify guidance documents related to antenatal oral healthcare. Quality of documents was appraised using the Appraisal of Guidelines Research and Evaluation II tool, and a 3-step quality cut-off value was used. Inductive thematic analysis was employed to categories discreet recommendations into themes.

**Results:**

Six guidelines and one consensus statement were analysed. Two documents developed within Australia scored ≥60% across five of the six domains of the quality appraisal tool and were recommended for use. Four documents (developed in the United States and Canada) were recommended for use with modifications, whilst one document (developed in Europe) was not recommended. A total of 98 discreet recommendations were identified and demonstrated considerable unanimity but differed in scope and level of information. The main content and number of recommendations were inductively categorised within the following clinical practice points: risk factor assessments (n = 2), screening and assessment (n = 10), pre-pregnancy care (referral, n = 1), antenatal care (health education and advice, n = 14; management of nausea and vomiting, n = 7; referral, n = 2), postnatal care (health education and advice, n = 1; anticipatory guidance, n = 6), documentation (n = 4), coordinated care (n = 4), capacity building (n = 6), and community engagement (n = 1).

**Conclusion:**

The methodological rigour of included guidance documents revealed areas of strengths and limitations and posit areas for improvement. Further research could centre on adapting antenatal oral healthcare guidelines and consensus statements to local contexts. More high-quality studies examining interventions within antenatal oral healthcare are needed to support the development of recommendations.

## Introduction

Oral health is a critical component of general health and wellbeing. Pregnancy signifies a unique and vulnerable period in a woman’s life and increases susceptibility to oral diseases such as periodontal disease and dental caries due to complex hormonal, behavioural, and physiologic changes [1]. Evidence has suggested an association between periodontal diseases and risk of adverse pregnancy outcomes during pregnancy, including preeclampsia, low-birthweight and preterm birth [2]. Other evidence has demonstrated the impact of poor oral health of women throughout the lifespan and that of their children, including the development of dental caries, impaired nutrition, increasing dental costs, and diminished quality of life [3]. These outcomes can be worse for women who are vulnerable, disadvantaged or Indigenous [1]. In light of the evidence, maintaining oral health during pregnancy has been continually recognised as a pressing public health concern worldwide [4], whilst oral healthcare topics relating to pregnancy have also been introduced into formal medical pedagogy [5], professional development training [6], and integrated primary healthcare models [7].

The role of antenatal care (ANC) providers in promoting oral health during pregnancy have emerged as a beneficial and cost-effective strategy to facilitate women accessing dental services and improving the oral health of women and their children [8]. As an umbrella term, ANC providers are healthcare professionals involved in antenatal care for women during pregnancy and include medical practitioners, obstetric specialists, midwives, nurses, and Indigenous healthcare workers. These providers are often the first point of contact for pregnant women and are well-placed to deliver oral health promotion and interventions prior to formal assessments by dental professionals.

Guidance documents, including clinical practice guidelines (CPGs) and consensus statements, can assist ANC providers and benefit women in promoting evidence-based practices. As defined by the Health and Medicine Division of the American National Academics (formerly the Institute of Medicine), CPGs are statements that “are informed by a systematic review of evidence and an assessment of the benefits and costs of alternative care options” [9]. In contrast, consensus statements are statements “developed by an independent panel of experts, usually multidisciplinary, convened to review the research literature for the purpose of advancing the understanding of an issue, procedure, or method” [10]. These terms are often used interchangeably and are both used to optimise patient care. Despite the existence of antenatal oral healthcare CPGs and consensus statements, considerable variation in rates of oral healthcare interventions exists among ANC providers who remain uncertain of their ability to implement recommendations [11–13]. It is possible that practice variation derives from discrepancies in recommendations, limited awareness of guideline existence and expert consensus, or differences in methodological quality during development [11, 14]. Moreover, it is often unknown whether recommendations are in accordance with best evidence and are suited to the local context [14]. This variation in the quality and content of CPGs and consensus statements addressing women’s oral health issues could result in conflicting recommendations making it challenging for ANC providers to deliver consistent quality health care.

Accordingly, evaluating the methodological quality and content of recommendations within guidance documents is imperative [15–17]. To our knowledge, a critical evaluation of antenatal CPGs and consensus statements for the management of oral health during pregnancy has not been previously conducted.

Therefore, the objectives of this systematic review were to:

1. Review the content of eligible antenatal oral healthcare CPGs and consensus statements; and,
2. Appraise the quality of methodology to inform areas of development, clinical practice, and research focus.

## Materials and methods

The authors developed a detailed study protocol according to the Preferred Reporting Items for Systematic Reviews and Meta-Analyses Protocols (PRISMA-P) [18]. The systematic review was reported according to the PRISMA statement [19].

### Eligibility criteria

The eligibility criteria were predetermined by the authors. Guidance documents were included if they were: (1) written in English; (2) published in developed countries for comparison according to the Organisation for Economic Co-operation and Development (countries that are regarded as developed countries due to their high Human Development Index and very high-income economies; see S1 Table); (3) labelled as CPG, guideline, consensus statement, recommendation, guidance statement, position paper, or professional standard; (4) allocated either entirely to antenatal oral healthcare or contained a minimum of two explicit recommendations on antenatal oral health; (5) published or updated between 2010 and 2020 as guidelines published prior to 2010 could be considered out of date and not reflect contemporary practices; (6) obtained from original sources (de novo development); and (7) most updated document if multiple versions existed. Documents were excluded if published earlier than 2010 or within a developing or least developed country, written in languages other than English, adapted from other sourced CPGs and consensus statements, or did not meet the definitions of CPGs or consensus statements as previously defined [9, 10].

### Search strategy

Three systematised search strategies were conducted to identify relevant guidelines and consensus statements between 16^th^ October 2020 to 23^rd^ October 2020. This approach involved only one reviewer (AW) due to the scope of the paper and resource constraints [20]. The three search strategies are outlined as follows:

1. A systematic search was conducted within five electronic databases: MEDLINE via PubMed, the Cumulative Index of Nursing and Allied Health Literature (CINAHL), Cochrane Library, Embase, and Scopus. Limiters by year (1^st^ Jan 2010 to 31^st^ Dec 2020) and language (English) were applied. MeSH terms including “pregnant women”, “prenatal care”, “oral health”, “practice guidelines”, “guidelines”, “consensus”, and “standard of care” and associated keywords were used.
2. A similar guideline review [21] has demonstrated sourcing guidance documents from grey literature sources as effective. Thus, an internet web search (in the Google search engine) using the terms “oral health”, and “guideline”, in conjunction with interchangeable terms of “antenatal”, “prenatal”, “perinatal”, “pregnancy”, and “maternal” were comprehensively examined up to the first 150 results, as these were considered most relevant.
3. Documents were purposively sought from prominent guideline development groups within eligible countries such as the Australian National Health and Medical Research Council (NHMRC), Guidelines International Network, Scottish Intercollegiate Guidelines Network, National Guideline Clearinghouse, and the National Institute of Health and Care Excellence. Databases of national and international professional societies related to the field of antenatal oral healthcare were also searched, such as the American College of Obstetricians and Gynaecologists, Royal College of Obstetricians and Gynaecologists, Royal College of Australian and New Zealand College of Obstetricians and Gynaecologists, and Society of Obstetricians and Gynaecologists of Canada, among others. The combined search term ‘pregnancy + oral health + guideline’ was used, and the first 20 results were examined for relevancy.

A secondary search using the Google search engine strategy to check for updated or additional sources was undertaken on 20^th^ April 2021 by the first author (AW), but no additional CPGs or consensus statements were identified. However, a 2020 updated version of an Australian guideline was identified and replaced an existing 2019 version within our review [22]. Detailed search processes with results are outlined in the S1 Appendix.

### Screening

Using EndNote X9.3.1 software (Clarivate Analytics, PA, USA), the first author (AW) conducted the literature searches, then screened potential results by title and abstract, retrieved the full-text document and excluded those that did not meet eligibility criteria. References from full-text documents were also cross-referenced by AW to assess for additional guidance documents. Results were periodically shared with the research team, and any disagreements were resolved by consensus via discussion among all five reviewers (AW, HH, HB, LC and SB). The reasons for excluding guidance documents were documented.

### Data extraction

One reviewer (AW) independently reviewed the guidance documents and extracted characteristic information relating to: title, development organisation, country/region, publication year, guidance document type (for example, whether evidence-based or based on expert consensus) and number of references. The content of recommendations within the guidance documents was extracted according to a predetermined recommendation extraction form by the authors and was adapted from the matrix developed by Zhang et al. [23] (See S2 Appendix). The following information was extracted from each document: title, author, publication year, guidance document type, funding, methodology, and relevant recommendations. Recommendations concerning antenatal oral healthcare were systematically extracted for further analysis and were repetitively and recursively analysed. We employed a thematic analysis using an inductive approach and categorised discrete recommendations by themes [24]. All extracted data were checked for accuracy by a second, third and fourth reviewer at random (HH, HB and SB).

### Quality appraisal

Three reviewers (HH, HB and SB) independently evaluated the quality of two or more guidance documents, and one reviewer (AW) independently evaluated the quality of all included guidance documents (until each document was appraised a minimum of twice). Disagreements were resolved through consensus discussion; when needed, a fifth reviewer (LC) participated in the discussion until agreement was achieved. We used the Appraisal of Guidelines for Research and Evaluation (AGREE) II tool [16], a validated and widely adopted tool that appraises methodological rigour and guideline development transparency. The AGREE II tool includes 23 items on a seven-point Likert scale across six domains. Each domain captures a distinct facet of guideline quality: ‘Scope and Purpose’ (n = 3), ‘Stakeholder Involvement’ (n = 3), ‘Rigour of Development’ (n = 8), ‘Clarity of Presentation’ (n = 3), ‘Applicability’ (n = 4) and ‘Editorial Independence’ (n = 2). An overall score of each domain was calculated as a percentage as follows: [total actual domain score–minimum possible domain score]/[maximum possible domain score–minimum possible domain score] x 100. Comments regarding the justification of scores, strengths and limitations were recorded into a separate table.

In addition, the AGREE II tool also included two overall quality assessments for each document: a final quality score of 1 to 7 and whether we would recommend using the document, categorising it as ‘recommend’, ‘recommend with modifications’, or ‘not recommend’. However, the AGREE II tool does not report a threshold for domain score quality, making it challenging to distinguish between high-, medium- and low-quality guidance documents. Following the 3-step system used in a similar review [25], we modified this approach to determine both overall guideline quality and recommendations for clinical use. Documents were deemed high-quality (recommended) if most domain scores (at least five of six) were greater than 60%, whilst documents were deemed medium-quality (recommended with modifications) if most domain scores were between 30–60% or at least two domain scores were no less than 60%. Documents were deemed low-quality (not recommended) if most of the domain scores were less than 30%.

### Statistical analysis

One reviewer (AW) performed statistical analyses using Stata version 17.0 (StataCorp, TX, USA). Descriptive statistics (mean, median, standard deviation [SD] and range) were calculated for each domain score. The intraclass coefficient (ICC) with its 95% confidence interval (95% CI) was calculated as an overall indicator of agreement on quality scores between reviewers. The degree of agreement between 0.01 and 0.20 is slight, from 0.21 to 0.40 is fair, from 0.41 and 0.60 is moderate, from 0.61 to 0.80 is substantial, and from 0.81 to 1.00 is almost perfect to perfect [26]. An independent *t*-test was undertaken to evaluate the differences between means of relevant variables, and *p*-values of 0.05 or less were considered significant.

## Results

### Search results

**Fig 1:**
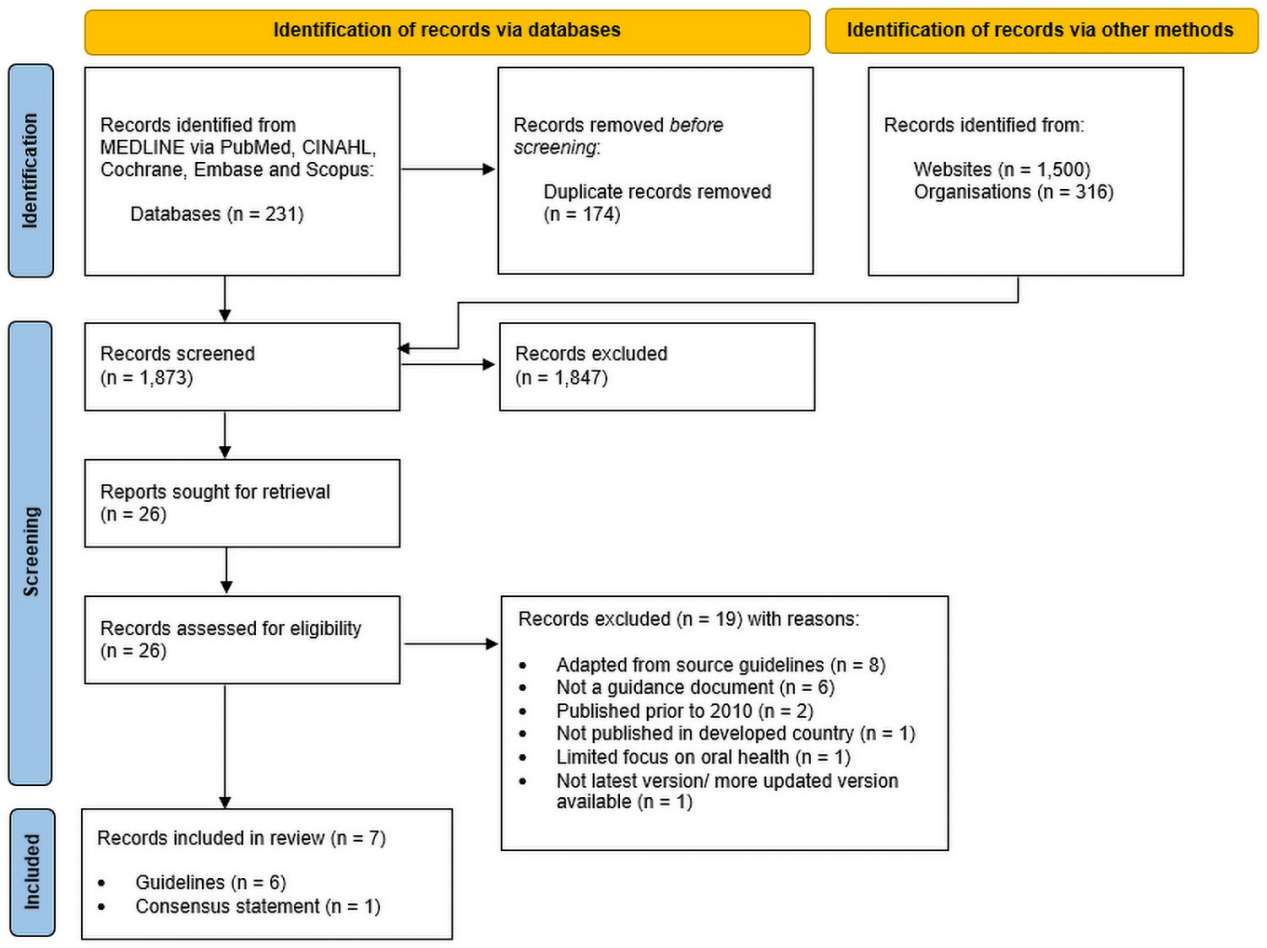
Flow chart of the systematic literature search and selection.

The search yielded 1,873 records after removing duplicates. After title and abstract screen, 26 records were obtained for full review; 19 were excluded based on: not satisfying our guidance document definitions (n = 5, factsheets [27–31]; n = 1, expert committee opinion [32]); published prior to 2010 (n = 2) [33, 34]; were not published in prespecified developed countries (n = 1, in Sri Lanka) [35]; provided a limited focus on antenatal oral healthcare (n = 1) [36]; were adapted from source CPGs or consensus statements included in our search (n = 8) [37–44]; or were not the latest version (n = 1) [45] (Fig 1). A final seven guidance documents (n = 6, CPGs; n = 1 consensus statement) met the eligibility criteria and were included in the review for analysis. Summary characteristics of included guidance documents are presented in Table 1.

**Table 1:** Summary characteristics of included guidance documents.

Title	Development organisation	Country/region	Publication year	Guidance document type	Number of references
Clinical Practice Guidelines: Pregnancy Care [22]	Australian Government Department of Health	Australia	2020	Evidence-based	27
National Guide to Preventive Health: Assessment for Aboriginal and Torres Strait Islander People [46]	National Aboriginal Community Controlled Health Organisation / the Royal Australian College of General Practitioners	Australia	2018	Evidence-based	13
Guidelines on Perinatal and Infant Oral Health Care [47]	American Academy of Pediatric Dentistry	United States	2016	Expert consensus	61
Oral Health During Pregnancy and Early Childhood: Evidence-based Guidelines for Health Professionals [48]	California Dental Association	United States	2010	Expert consensus	249
Oral Health During pregnancy: A National Consensus Statement [49]	Oral Health Care During Pregnancy Expert Workgroup	United States	2012	Expert consensus	None clearly provided within the guideline.
Provincial Perinatal Guidelines: Population and Public Health Prenatal Care Pathway [50]	Perinatal Services British Columbia	Canada	2014	Expert consensus	3
The Relationship Between Oral Health and Pregnancy: Guidelines for Non-dentistry Health Professionals [51]	European Federation of Periodontology	Europe	2020	Expert consensus	None clearly provided within the guideline.

### Characteristics of included guidance documents

Guidance documents were identified and were published by the following organisations: American Academy of Pediatric Dentistry (AAPD) [47], Australian Government Department of Health (AGDH) [22], California Dental Association Foundation (CDAF) [48], European Federation of Periodontology (EFP) [51], National Aboriginal Community Controlled Health Organisation/The Royal Australian College of General Practitioners (NACCHO/RACGP) [46], Oral Health Care During Pregnancy Expert Workgroup (OHCDPEW) [49], and Perinatal Services British Columbia (PSBC) [50]. Six guidelines (AAPD, AGDH, CDAF, EFP, NACCHO/RACGP and PSBC) and one consensus statement (OHCDPEW) were included. These guidance documents were developed from three continents including North America (the United States [US] [n = 3, AAPD, CDAF and OHCDPEW] and Canada [n = 1, PSBC]), Australia (n = 2, AGDH and NACCHO/RACGP), and Europe (n = 1, EFP). Most documents were developed for implementation at a national level (n = 5, AAPD, AGDH, CDAF, NACCHO/RACGP and OHCDPEW), state or province levels (n = 1, PSBC) or international level (n = 1, EFP). The types of documents were based on expert consensus (n = 5, AAPD, CDAF, EFP, OHCDPEW and PSBC) or evidence-based methodology (n = 2, AGDH and NACCHO/RACGP). The number of references included within the guidance documents varied (range 3 to 249; AAPD, AGDH, CDAF, NACCHO/RACGP and PSBC). However, two documents (EFP and OHCDPEW) did not clearly provide references.

Most guidance documents were exclusively developed for antenatal oral healthcare and provided comprehensive recommendations (n = 4, AAPD, CDAF, EFP and OHCDPEW), while other documents were developed for general aspects of antenatal care and included a section or chapter of oral healthcare recommendations (n = 4, AGDH, NACCHO/RACGP and PSBC). External funding from academic institutions, government and non-government entities (n = 9, AGDH, CDAF, OHCDPEW, NACCHO/RACGP), and a consumer goods corporation (n = 1, EFP) were disclosed in several documents. Only two documents developed by the AGDH and NACCHO/RACGP provided a level of evidence and grading of recommendations using the NHMRC system [52].

### Summary of recommendations

All relevant guidance document information and recommendations were extracted using the recommendation extraction form (S2 Appendix). A total of 98 discreet recommendations were identified. Of these 98 recommendations, two were based on the systematic assessment of evidence, and the remaining 96 were based on expert consensus. The two evidence-based recommendations are outlined in S2 Table. These recommendations received an overall grading of recommendation of ‘B’, indicating that the body of evidence can be trusted to guide practice in most situations. Overall, oral healthcare recommendations and management options demonstrated considerable unanimity but differed in scope and level of information. The main content and number of recommendations have been inductively categorised within the following clinical practice points: risk factor assessments (n = 2), screening and assessment (n = 10), pre-pregnancy care (referral, n = 1), antenatal care (health education and advice, n = 14; management of nausea and vomiting, n = 7; referral, n = 2), postnatal care (health education and advice, n = 1; anticipatory guidance, n = 6), documentation (n = 4), coordinated care (n = 4), capacity building (n = 6), and community engagement (n = 1), and are presented in Table 2.

**Table 2:** Summary of key recommendations within included guidance documents.

Clinical practice point	Example recommendation and guidance document
**Risks factor assessment**	Encourage all women at the first prenatal visit to schedule a dental examination if one has not been performed in the past six months, or if a new condition has developed or is suspected (CDAF).
	If the last dental visit took place more than 6 months ago or if any oral health problems were identified during the assessment, advise women to schedule an appointment with a dentist as soon as possible (OHCDPEW).
**Screening and assessment**	Ask the woman if she has any concerns/fears about getting dental care while pregnant. Based on her response, be ready to inform her that dental care is safe during pregnancy and address specific concerns (CDAF).
	As a routine part of the initial prenatal examination, conduct an oral health assessment of the teeth, gums, tongue, palate and mucosa (CDAF).
	Health professionals should include an oral-health screening, oral health history and examination as part of their regular medical examination (EFP).
	At the first antenatal visit, undertake an oral health review including the assessment of teeth, gums and oral mucosa, as part of a regular health check (NACCHO/RACGP).
	Visually inspect teeth for evidence of caries, periodontal disease, assessment of maternal caries and/or poor oral hygiene (NACCHO/RACGP).
	Assess oral hygiene practices and consumption of sucrose and sweetened drinks, especially in baby bottles, ‘honey on the dummy’ or other sweet substances such as glycerine on the dummy, and intake of sugared medicines (NACCHO/RACGP).
	Assess access to fluoridated water supply advice (NACCHO/RACGP).
	During the initial prenatal evaluation, take an oral health history and check the mouth for problems such as swollen or bleeding gums, untreated dental decay, mucosal lesions, signs of infection, or trauma (OHCDPEW).
	Screen women for concerns related to oral health and access to oral health care (PSBC).
	Assess woman’s knowledge related to recommended oral health care during pregnancy and her ability to access dental health care (PSBC).
**Pre-pregnancy care**	
Referral	Health professionals who treat women who want to become pregnant should also recommend that their patients visit an oral-health professional and establish healthy periodontal conditions before pregnancy, because this may favour the outcome of the planned pregnancy (EFP).
**Antenatal care**	
Health education and advice	Inform women that dental treatment during pregnancy, including dental radiographs with proper shielding and local anaesthetic, is safe in all trimesters and optimal in the second trimester (AAPD).
	Educate women on proper oral hygiene, using a fluoridated toothpaste, chewing sugar-free gum, and eating small amounts of nutritious food throughout the day to help minimise their caries risk (AAPD).
	At the first antenatal visit, advise women to have oral health checks and treatment, if required as good oral health is important a woman’s health and treatment can be safely provided during pregnancy (AGDH).
	Educate the pregnant woman about the importance of her oral health, not only for her overall health but also for the oral health of her children (CDAF).
	Advise the pregnant woman that prevention, diagnosis and treatment of oral diseases are highly beneficial and can be undertaken any time during pregnancy with no additional foetal or maternal risk as compared to not providing care (CDAF).
	Inform the pregnant woman that dental care can improve her overall health and the health of her developing foetus and her children (CDAF).
	Educate women and encourage behaviours and oral hygiene measures that support good oral health (CDAF).
	Oral health education: As part of their regular care, health professionals should provide oral-health education and oral-health screening to pregnant women (EFP)
	At the first antenatal visit, advise women to have an oral health check and treatment if required (NACCHO/RACGP).
	Advise about smoking cessation and limiting alcohol consumption (NACCHO/RACGP).
	Advise about the hazards of high carbohydrate and acidic snacks and drinks taken between meals (NACCHO/RACGP).
	Advise against high and regular consumption of black cola, sweetened fizzy drinks and sports drinks, with water being the preferred drink (NACCHO/RACGP).
	Advise pregnant women about oral health care: Reassure women that oral health care, including use of radiographs, pain medication, and local anaesthesia, is safe throughout pregnancy (OHCDPEW).
	Offer women information about the importance of oral health in pregnancy and about how and where they can access dental health services (PSBC).
Management	Provide advice on oral health to women who experience nausea and vomiting: Explain that vomiting exposes teeth to acid and give tips on how to reduce the impact (AGDH).
	Eat small amounts of nutritious yet noncariogenic foods—snacks rich in protein, such as cheese—throughout the day (CDAF).
	Use a teaspoon of baking soda (sodium bicarbonate) in a cup of water to rinse and spit after vomiting, avoiding tooth brushing directly after vomiting as the effect of erosion can be exacerbated by brushing an already demineralised tooth surface (CDAF).
	Use gentle tooth brushing and fluoride toothpaste twice daily to prevent damage to demineralised tooth surfaces (CDAF).
	Use a fluoride-containing mouth rinse immediately before bedtime to help remineralise teeth (CDAF).
	Brush teeth twice daily with a soft toothbrush and fluoride toothpaste and advise to spit, not rinse, excess paste (NACCHO/RACGP).
	Women experiencing vomiting in pregnancy (“morning sickness”) should avoid brushing for an hour after vomiting to protect tooth enamel but can rinse their mouths with water or fluoride mouth wash (PSBC).
Referral	If urgent care is needed, write and facilitate a formal referral to a dentist who maintains a collaborative relationship with the prenatal care health professional (OHCDPEW).
	Refer woman to local dental health professionals as indicated (PSBC).
**Postnatal care**	
Health education and advice	Advise and encourage the woman to obtain necessary follow-up dental care and oral health maintenance during the postpartum period and thereafter (CDAF).
Anticipatory guidance	Educate women regarding her diet including the adequate quality and quantity of nutrients for the mother-to-be and the child. This education also should include information regarding the caries process and food cravings that may increase the mother’s caries risk (AAPD).
	Parents should be encouraged to establish a dental home for infants by 12 months of age (AAPD).
	Advise women on actions that may reduce the risk of caries in their children (CDAF).
	Encourage and support a woman’s decision to breastfeed, providing appropriate oral hygiene instructions for after feeding, and have ready access to resources (CDAF).
	Promote breastfeeding, with weaning to a baby cup, not a bottle. If bottles are used, advise against the use of any fluid apart from water and do not put baby to sleep with a bottle (NACCHO/RACGP).
	Advise woman that oral health care is important for the prevention of tooth decay, periodontal disease and to prevent transmission of oral bacteria that may cause tooth decay for her child. Women should brush with a fluoride toothpaste at least twice daily and floss daily (PSBC).
**Documentation**	Determine and document in the prenatal record oral health findings and whether the patient is already under the care of an oral health professional (CDAF).
	Facilitate dental care by providing written consultation or an oral health referral form (CDAF).
	Document your findings in the woman’s medical record (OHCPDEW).
	On the patient-intake form, include questions about oral health (OHCDPEW).
**Coordinated care**	Share appropriate clinical information with the oral health professional and answer questions that the oral health professional may ask about a patient or condition (CDAF).
	Establish relationships with oral health professionals in the community. Develop a formal referral process whereby the oral health professional agrees to see the referred individual in a timely manner and to provide subsequent care (OHCDPEW).
	Share pertinent information about pregnant women with oral health professionals, and coordinate care with oral health professionals as appropriate (OHCDPEW).
	Communicate and collaborate with the local resources to facilitate access to dental care for women with barriers (PSBC).
**Capacity building**	Provide education and dental referrals for oral health care, understanding that such care may have relatively low priority for some women, particularly those challenged by financial worries, unemployment, housing, intimate partner violence, substance abuse or other life-stressors (CDAF).
	Encourage women to learn more about oral health during pregnancy and early childhood by accessing available consumer information including reputable web sites (CDAF).
	Counsel women to follow oral health professionals’ recommendations for achieving and maintaining optimal oral health (OHCDPEW).
	Encourage women to seek oral health care, practice good oral hygiene, eat healthy foods, and attend prenatal classes during pregnancy (OHCDPEW).
	Provide support services (case management) to pregnant women (OHCDPEW).
	Support and assist a vulnerable woman and those needing help due to barriers or lack of skills to address oral health concerns, including referral to dental health providers, and supporting her to access care. Support woman to build knowledge and capacity to manage life-long oral health promoting habits for herself and her family (PSBC).
**Community engagement**	Establish partnerships with community-based programs that serve pregnant women with low incomes (OHCDPEW).

### Quality of included guidance documents

The overall agreement among reviewers of guidance documents against the AGREE II tool was almost perfect (overall ICC: 0.856; 95% CI: 0.710, 0.918), whilst the level of agreement between individual reviewers ranged from substantial to almost perfect (ICC: 0. 636–0.913). The ICC scores and their 95% CIs are reported in Table 3.

**Table 3:** Results of the intraclass correlation coefficient analysis of included guidance documents.

Development organisation	Intraclass correlation coefficient	95% Confidence interval
Australian Government Department of Health [22]	0.819	[0.577, 0.923]
National Aboriginal Community Controlled Health Organisation / the Royal Australian College of General Practitioners [52]	0.729	[0.286, 0.891]
American Academy of Pediatric Dentistry [47]	0.636	[-0.101, 0.866]
California Dental Association [48]	0.760	[0.259, 0.909]
Oral Health Care During Pregnancy Expert Workgroup [49]	0.853	[0.651, 0.938]
Perinatal Services British Columbia [50]	0.841	[0.448, 0.942]
European Federation of Periodontology [51]	0.913	[0.669, 0.969]

No documents scored ≥60% across all domains. Two documents scored ≥60% across five domains (n = 2, AGDH and NACCHO/RACGP), three documents scored between 30–60% across two or more domains (n = 4, AAPD, CDAF, OHCDPEW AND PSBC), whilst one document scored <60% across all domains (n = 1, EFP). The highest average domain score was ‘Scope and Purpose’ (79.3%, SD 19.4%), followed by ‘Clarity of Presentation’ (75.8%, SD 13.7%), ‘Stakeholder Involvement’ (59.9%, SD 23.5%), ‘Rigour of Development’ (49.8%, SD 39.9%), ‘Editorial Independence’ (30.9%, SD 40.1%), and ‘Applicability’ (27.9%, SD 20.2%). Notably, two evidence-based documents (46.2%, SD 23.9%; AGDH and NACCHO/RACGP) when compared to five documents based on expert consensus (82.5%, SD 17.1%; AAPD, CDAF, EFP, OHCDPEW and PSBC) scored significantly higher across domain scores (*t*[degrees of freedom] = *t* statistic, *p* = *p*-value: *t*[10] = 3, *p* = 0.013). However, no significant difference in domain scores were demonstrated when guidelines (56.0%, SD 23.6%; n = 5, AAPD, AGDH, CDAF, EFP, NACCHO/RACGP and PSBC) and consensus statement (41.7%, SD 22.7%; n = 1, OHCDPEW) were compared (*t*[10] = 1, *p* = 0.311). Guidance documents developed by the NACCHO/RACGP and the AGDH were deemed high-quality and recommended for use in clinical practice (n = 2), four documents were medium-quality and recommended with modifications (n = 5, AAPD, CDAF, OHCDPEW and PSBC), and one document (n = 1, EFP) was deemed low-quality and not recommended for use in clinical practice (See Table 4).

**Table 4:** AGREE II scores of included guidance documents.

Guideline organisation	Domain 1: Scope and purpose	Domain 2: Stakeholder involvement	Domain 3: Rigour of development	Domain 4: Clarity of presentation	Domain 5: Applicability	Domain 6: Editorial independence	Overall assessment* and recommendation†	Overall quality†
	%	%	%	%	%	%		
National Aboriginal Community Controlled Health Organisation / the Royal Australian College of General Practitioners [52]	94.4	91.7	92.7	91.7	47.9	83.3	7: Recommend	High
Australian Government Department of Health [22]	83.3	86.1	91.7	91.7	47.9	87.5	7: Recommend	High
California Dental Association [48]	97.2	61.1	60.4	75.0	31.3	4.2	5: Recommend with modifications	Medium
American Academy of Pediatric Dentistry [47]	86.1	50.0	60.4	69.4	45.8	0	4: Recommend with modifications	Medium
Perinatal Services British Columbia [50]	88.9	50.0	22.9	83.3	12.5	0	3: Recommend with modifications	Medium
Oral Health Care During Pregnancy Expert Workgroup [49]	61.1	58.3	17.7	61.1	10.4	41.7	3: Recommend with modifications	Medium
European Federation of Periodontology [51]	44.4	22.2	3.1	58.3	0	0	1: Not recommend	Low
Mean	79.3	59.9	49.8	75.8	27.9	30.9		
Median	86.1	58.3	60.4	75.0	31.3	4.2		
SD	19.4	23.5	39.9	13.7	20.2	40.1		
Range	44.4–97.2	22.2–91.7	3.1–92.7	58.3–91.7	0–47.9	0–87.5		

* Overall assessment based on final quality score between 1 and 7 from AGREE II tool.

^†^ Overall recommendation: documents were recommended if most domain scores (at least four of six) were greater than 60%; documents were recommended with modifications if most domain scores were between 30–60% or at least two domain scores were no less than 60%; documents were not recommended if most of the domain scores were less than 30%.

**Fig 2:**
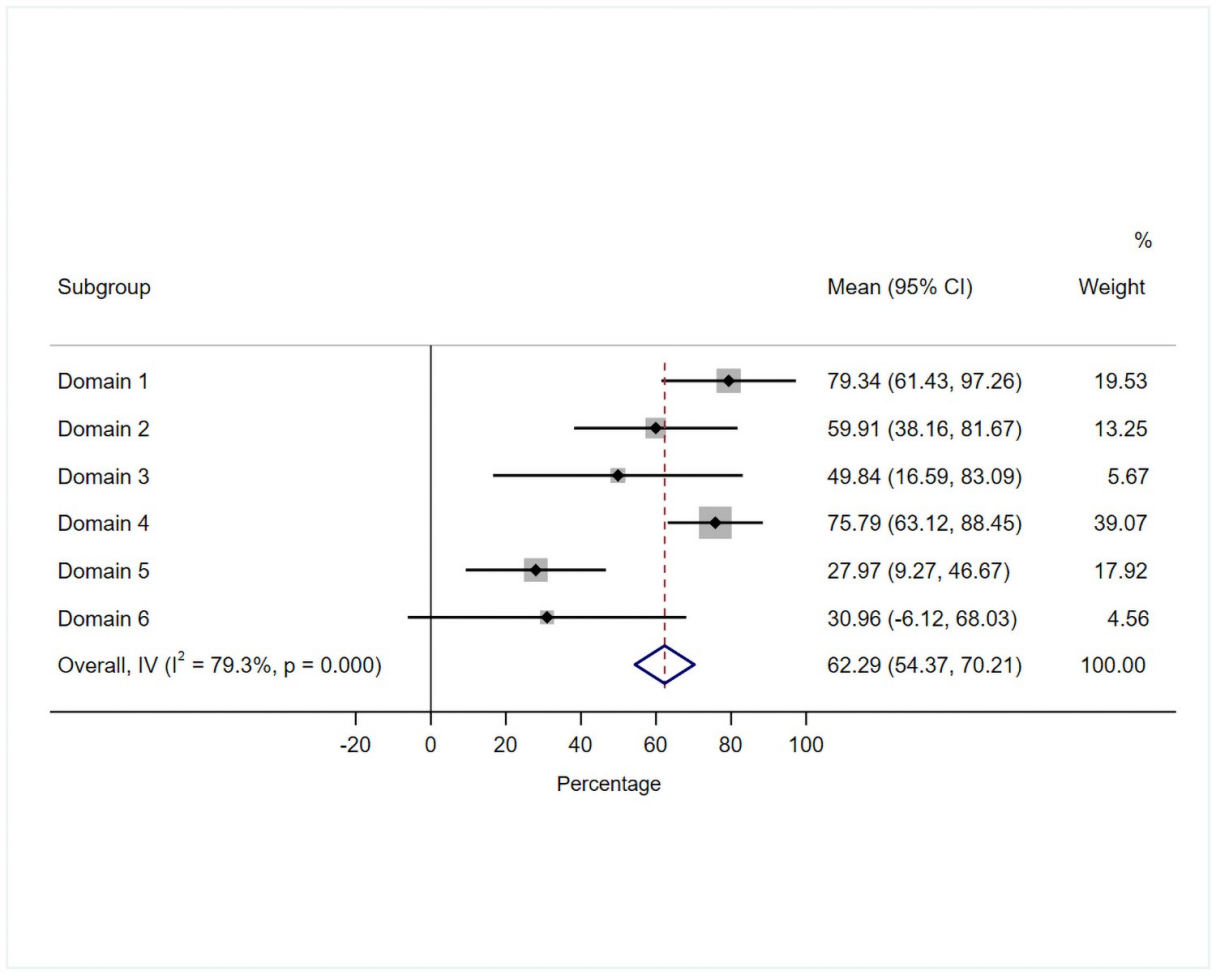
Mean standardised AGREE II domain scores of included guidance documents using a forest plot.

The standardised AGREE II domain scores of included guidance documents are presented as a forest plot (Fig 2), visually demonstrating areas of relative methodological strengths and weaknesses.

The strengths and limitations of included guidance documents based on the AGREE II criteria are summarised in Table 5 and are based on the consensus of our comments during the quality appraisal phase and highlight areas of potential improvement.

**Table 5:** Strengths and limitations of included guidance documents according to AGREE II criteria.

AGREE II Domain	Strengths	Limitations
**Domain 1**. Scope and purpose	Overall objectives of guidelines and intended use in management of pregnant women are clearly described (AAPD, AGDH, CDAF, EFP, NACCHO/RACGP, OHCDPEW, PSBC).	Health questions are not clearly described and/or lack specific oral health-related questions in its methodology (AAPD, CDAF, EFP, NACCHO/RACGP, OHCDPEW, PSBC).
**Domain 2**. Stakeholder involvement	Names, specialties, institutions, and geographical locations of relevant professional groups were clearly mentioned and easy to find (AGDH, CDAF, NACCHO/RACGP, OHCDPEW, PSBC).	Guideline development groups not clearly defined or difficult to find (AAPD, EFP).
	Target users clearly defined (AGDH, CDAF, NACCHO/RACGP, OHCDPEW, PSBC).	Lack of adequate and clear involvement of pregnant women within its development (AAPD, CDAF, EFP, OHCDPEW, PSBC).
	Included women and pregnant women representatives (AGDH, NACCHO/RACGP).	
**Domain 3**. Rigour of development	Mentioned a detailed search strategy of supporting evidence (AAPD, AGDH, NACCHO/RACGP).	Lacked a detailed search strategy (CDAF, EFP, OHCDPEW, PSBC).
	Utilised a quality grade of recommendation and evidence system in developing recommendations (AGDH, NACCHO/RACGP).	Quality assessment of evidence and its limitations not clearly reported (AAPD, CDAF, EFP, OHCDPEW, PSBC).
	Formulation of recommendations include detailed discussion on health benefits and risks (AGDH, NACCHO/RACGP).	Lack detailed discussion of benefits and risks in formulating recommendations (AAPD, CDAF, EFP, OHCDPEW, PSBC).
	Development of recommendations and their links to supporting evidence clearly defined (AGDH, NACCHO/RACGP).	Review and update processes not defined (AAPD, CDAF, EFP, OHCDPEW, PSBC).
	Guideline was externally reviewed by experts (AAPD, AGDH, CDAF, EFP, NACCHO/RACGP, OHCDPEW, PSBC).	
	Updating and review processes clearly outlined (AGDH, NACCHO/RACGP).	
**Domain 4**. Clarity and presentation	Key recommendations were specific, unambiguous and easily identifiable (AAPD, AGDH, CDAF, NACCHO/RACGP, OHCDPEW, PSBC).	Key recommendations targeted to users slightly ambiguous (EFP).
		Management of nausea and vomiting during pregnancy not mentioned (AAPD, EFP, OHCDPEW).
**Domain 5**. Applicability	Discussed facilitators and barriers to implementation (AGDH, NACCHO/RACGP).	Facilitators and barriers to implementation not explicitly discussed (AAPD, CDAF, EFP, OHCDPEW, PSBC).
	Provided implementation tools including educational resources, protocols, summary documents, patient information, assessment and questionnaire forms, or clinical pathway processes (AGDH, CDAF, EFP, NACCHO/RACGP, OHCDPEW, PSBC).	Advice and tools on implementation not provided (AAPD).
		Quality measures and indicators on monitoring and clinical auditing not clearly reported (AAPD, AGDH, CDAF, EFP, NACCHO/RACGP, OHCDPEW, PSBC).
		Lacked formal economic analysis that was easily identifiable (AAPD, AGDH, CDAF, EFP, NACCHO/RACGP, OHCDPEW, PSBC).
**Domain 6**. Editorial independence	Funding and influence statement was reported (AGDH, NACCHO/RACGP, OHCDPEW).	Influence of funding not clearly reported (AAPD, CDAF, EFP, PSBC).
	Competing interests were clearly provided (AGDH, NACCHO/RACGP).	Competing interests of guideline developers not explicitly provided (AAPD, CDAF, EFP, OHCDPEW, PSBC).

## Discussion

To the best of our knowledge, this is the first study to critically evaluate the quality and content of antenatal CPGs and consensus statements relating to oral healthcare during pregnancy within developed countries. Seven antenatal oral healthcare guidance documents (six guidelines and one consensus statement) were identified and were appraised using the AGREE II tool. Our findings highlighted several areas for improvement and development, clinical practice, and research focus.

The guidance documents developed by AGDH and NACCHO/RACGP scored ≥60% across five domains of the AGREE II tool, were deemed high-quality and were recommended for use in clinical practice. We attributed this to the robust and transparent methods reported by the guideline developers. This finding was further supported when guidance documents based on evidence-based methodology (n = 2, AGDH and NACCHO/RACGP) demonstrated significantly higher quality scores across domains when compared to guidance documents based on expert consensus (n = 5, AAPD, CDAF, EFP, OHCDPEW and PSBC). None scored ≥60% across all domains, whilst four documents developed by APPD, CDAF, OHCDPEW and PSBC were deemed medium-quality and recommended with modifications and posited areas for improvement. One document (EFP) did not achieve our cut-off value of 60% within any domains, was deemed low-quality and was not recommended for use in clinical practice, suggesting significant room for improvement in its methodology. To illustrate, all documents demonstrated a need to improve their applicability (Domain 5), especially in areas concerning the discussion of facilitators and barriers to implementation, inclusion of quality measures and indicators to monitoring and clinical audit, and evidence of economic analysis. In another example, five documents (AAPD, CDAF, EFP, OHCDPEW and PSBC) did not indicate a disclosure of competing interests statements highlighting a need to improve their editorial independence (Domain 6). Four documents (AAPD, OHCDPEW, EFP and PSBC) also demonstrated deficiencies within stakeholder involvement (Domain 2), most notably in the lack of pregnant women involvement during development stages. Whilst three documents (EFP, OHCDPEW and PSBC) had significant gaps in their rigour of development (Domain 3), including lack of systematic literature search, quality assessment of the evidence, detailed discussion of benefits and risks in formulating recommendations, and reporting of review and update processes. Our lowest scoring domains of editorial independence (Domain 6) and applicability (Domain 5) align with findings from a systematic review of 42 appraisal studies, including 626 CPGs across several disciplines [53].

However, in spite of the variations in methodological rigour, the overall oral healthcare recommendations across guidance documents were consistent and included risk factor assessments, screening and assessment, pre-pregnancy care (referrals), antenatal care (health education and advice, management of nausea and vomiting, and referrals), postnatal care (health education and advice and anticipatory guidance), documentation, coordinated care, capacity building, and community engagement as it related to the management of oral health during pregnancy. Only recommendations for ANC providers to advise women to have an oral health check at the initial antenatal visit were supported by a level of evidence and grading of recommendations system [52]. Overall, it appears that the assessment of current evidence evaluating oral healthcare interventions provided by ANC providers is relatively scant within the literature. To illustrate, we could only easily locate four systematic reviews [13, 54–56] and one scoping review [57] relating to this topic published within the past decade. Therefore, more high-quality studies using rigorous methodologies are needed to support the development of recommendations concerning antenatal oral healthcare.

Our systematic assessment of antenatal oral healthcare guidance documents could be beneficial in supporting the decision to adopt or adapt guidance documents into clinical practice or within different local contexts. For guidance documents to be considered trustworthy and of high quality, the following criteria from the NHMRC Standards for Guidelines is suggested: (1) guidance documents should be relevant and useful for decision-making; (2) be transparent; (3) be overseen by a guideline development group; (4) identify and manage conflicts of interest; (5) be focused on health and related outcomes; (6) be evidence-informed; (7) make actionable recommendations; (8) be up-to-date; and (9) be accessible [52]. Based on these criteria and because of the high methodological rigour within the CPGs developed by AGDH and NACCHO/RACGP, we consider the adoption of their recommendations as appropriate and indicative of the best available evidence. The adaptation of CPGs and consensus statements has been acknowledged as a valid and less resource-intensive alternative to de novo development [58]. Within antenatal oral healthcare, several adapted guidelines have been published within the past ten years, particularly within the US [37–40, 42–44] and Canada [41]. Though beyond the scope of our systematic review, the option to adapt rather than develop guidance documents could prove particularly advantageous to developing and least developed countries and could implement existing formal adaptation methodologies and frameworks [59, 60]. Most notably, guidance adaptation and knowledge synthesis within antenatal oral healthcare, both within developed, developing and least developed countries, signifies a dearth of literature and suggests an area for future research.

Our critical appraisal of antenatal oral healthcare guidance documents further highlights the flexibility and diverse use of the AGREE II tool to evaluate methodological rigour appropriately and effectively. Within 2021, systematic reviews of pregnancy-related guidance documents using the AGREE II tool have included gestational weight management [61], gestational diabetes [62], use of complementary medicines and therapies in antenatal care [63], and prevention of preeclampsia [64], among other related topics. These reviews highlighted gaps in methodology and guideline development, consistencies and inconsistencies within recommendations and management options, and provided suggestions for improvements.

### Strengths and limitations

This is the first systematic review to appraise guidance documents on antenatal oral healthcare and identify and synthesise the content of recommendations. Systematic methods in the review processes and quality appraisal were performed with the AGREE II tool, which remains a well-established and validated instrument. Our adapted version of the ‘recommendation matrix’ developed by Zhang et al. [23] proved a useful and systematic data extraction method. Overall, the findings of our review have the potential to provide pragmatic guidance on areas of antenatal oral healthcare, particularly as it pertains to the methodology of recommendation development, clinical practices of ANC providers, and the identification of gaps in areas requiring further research.

However, this systematic review had several limitations. The purposive search of guidance documents was limited to professional and guideline development groups within developed and English-speaking countries. Sources from less developed and non-English speaking countries were thus likely overlooked and could limit our findings’ generalisability and relevance to local contexts and healthcare systems. In addition, the AGREE II tool was originally designed to evaluate CPGs [16]. Despite opinions within the literature that consensus statements should be subjected to the same rigorous appraisal methods for their development as CPGs [65, 66], it may be important to consider this limitation when interpreting our findings for the consensus statement developed by the OHCDPEW [49]. Notwithstanding, the consensus statement developed by the OHCDPEW did not demonstrate a significant difference in quality when compared to the six eligible guidelines within our review, which may add some credence to the diverse application of the AGREE II tool.

As an inherent limitation when conducting a systematic review, a potential for reviewer bias exists. However, the level of agreement using the AGREE II tool was substantial to almost perfect among reviewers, suggesting that we were relatively unanimous in our interpretation of quality.

## Conclusions

The methodological qualities of seven antenatal oral healthcare guidance documents within developed countries were appraised and revealed areas of strengths and limitations. Guidance documents developed by the AGDH and NACCHO/RACGP presented the highest methodological rigour, were developed using an evidence-based methodology and were recommended for use in clinical practice. The content of recommendations was relatively consistent but differed in scope and level of information. Further research could centre on adapting existing antenatal oral healthcare CPGs and consensus statements to local contexts. More high-quality studies examining interventions within antenatal oral healthcare are needed to support development of recommendations.

## Supporting information

S1 Appendix Detailed search strategy and results. (DOCX)

S2 Appendix Recommendation extraction forms of included guidance documents. (DOCX)

S1 Table List of organisations for economic co-operation and development member countries. As of 10.2020.
(DOCX)

S2 Table Grading of recommendations in evidence-based guidelines (n = 2). (DOCX)

S1 Checklist PRISMA 2009 checklist. (DOC)

S1 File List of relevant professional society websites. (DOCX)
