# Supplementary material for: Clinical practice guidelines and consensus statements for antenatal oral healthcare: An assessment of their methodological quality and content of recommendations
Source: PLoS One. 2022 Feb 3;17(2):e0263444. doi: 10.1371/journal.pone.0263444 (PMC8812839; doi:10.1371/journal.pone.0263444)
Supplement: S1 Appendix — (DOCX) [file pone.0263444.s001.docx]

**S1 APPENDIX**

**Detailed search strategy and results**

ELECTRONIC DATABASE SEARCH STRATEGY

Records were reviewed and managed within Endnote X9.3.1. Reference list of total records obtained for full-text review (n=57) were also screened for additional relevant sources.

| **Table S1A.** Electronic database search strategy and results. | | | |
| --- | --- | --- | --- |
| **Database** | **Step** | **Search strategy & key words** | **Results (16/10/2020)** |
| **MEDLINE via PubMed** | #1 | (((((((perinatal[Title]) OR (prenatal[Title])) OR (antenatal[Title])) OR (maternal[Title])) OR (pregnanc*[Title])) OR (obstetric[Title])) OR ("pregnant women"[MeSH Terms])) OR ("prenatal care"[MeSH Terms]) | 355303 |
|  | #2 | ((((((((((("dental assessment"[Title]) OR ("oral disease*"[Title])) OR ("oral diagnosis"[Title])) OR ("oral examination"[Title])) OR ("oral health assessment"[Title])) OR ("oral health care"[Title]) ) OR ("oral health counsel*"[Title])) OR ("oral health practice*"[Title])) OR ("oral health promotion"[Title])) OR ("oral health status"[Title])) OR ("oral health screening"[Title]) OR ("oral health"[MeSH Terms]) | 19615 |
|  | #3 | ((((((((((guideline*[Title]) OR (guidance*[Title])) OR (recommendation*[Title])) OR (consensus*[Title])) OR ("best practice*"[Title])) OR (statement*[Title])) OR (standard*[Title])) OR ("practice parameter*"[Title])) OR ("position paper*"[Title])) OR ("position stand*"[Title])) OR ("position statement*"[Title]) OR "guideline"[Publication Type] OR "guidelines as topic"[MeSH Terms] OR "Practice Guidelines as topic"[MeSH Terms] OR "Practice Guideline" [Publication Type] OR "consensus"[MeSH Terms] OR "standard of care"[MeSH Terms] | 397376 |
|  | #4 | #1 AND #2 AND #3 AND #4 | 23 |
|  | #5 | #4 Filters: Date: 1/1/2010 – 31/12/2020, English. | 15 |
| **Cumulative** **Index for Nursing and Allied Health Literature (CINAHL)** | #1 | TI perinatal OR TI prenatal OR TI antenatal OR TI maternal OR TI pregnanc* OR TI obstetric OR MH "prenatal care" OR MH "antenatal care" OR MH "perinatal care" | 120779 |
|  | #2 | TI "dental assessment" OR TI "oral disease*" OR TI "oral diagnosis" OR TI "oral examination" OR TI "oral health assessment" OR TI "oral health care" OR TI "oral health counsel*" OR TI "oral health practice*" OR TI "oral health promotion" OR TI "oral health status" OR TI "oral health screening" OR MH "oral health" | 13480 |
|  | #3 | TI guideline* OR TI guidance* OR TI recommendation* OR TI consensus* OR TI "best practice*" OR TI statement* OR TI standard* OR TI "practice parameter*" OR TI "position paper*" OR TI "position stand*" OR TI "position statement*" OR MH guideline* | 142782 |
|  | #4 | #1 AND #2 AND #3 | 11 |
|  | #5 | #4 Filters: Date: 1/1/2010 - 31/12/2020, English. | 9 |
| **Cochrane Library** | #1 | MeSH descriptor: [Guidelines as Topic] explode all trees | 1938 |
|  | #2 | MeSH descriptor: [prenatal care] explode all trees | 1579 |
|  | #3 | MeSH descriptor: [oral health] explode all trees | 461 |
|  | #4 | MeSH descriptor: [Consensus] explode all trees | 59 |
|  | #5 | perinatal):ti,ab,kw OR (prenatal):ti,ab,kw OR (antenatal):ti,ab,kw OR (obstetric):ti,ab,kw OR (pregnanc*):ti,ab,kw | 47569 |
|  | #6 | ("dental assessment"):ti,ab,kw OR ("oral disease*"):ti,ab,kw OR ("oral assessment"):ti,ab,kw OR ("oral health"):ti,ab,kw OR ("oral health care"):ti,ab,kw | 3767 |
|  | #7 | (guideline*):ti,ab,kw OR (guidance*):ti,ab,kw OR (recommendation*):ti,ab,kw OR (consense*):ti,ab,kw OR ("best practice*"):ti,ab,kw | 73619 |
|  | #8 | (statement*):ti,ab,kw OR (standard*):ti,ab,kw OR ("practice parameter*"):ti,ab,kw OR ("position paper"):ti,ab,kw OR ("position statement*"):ti,ab,kw | 225496 |
|  | #9 | #1 AND #2 AND #3 AND #4 AND #5 AND #6 AND #7 AND #8 | 0 |
|  | #10 | #5 AND #6 AND #7 AND #8 | 9 |
|  | #11 | Filters: Date: 1/1/2010 – 31/12/2020, Language (Not applicable). | 8 |
| **Embase** | #1 | ("prenatal care" or prenatal or antenatal or "antenatal care" or perinatal or pregnanc* or obstetric).ti. | 308302 |
|  | #2 | ("oral health" or "dental assessment" or "oral disease*" or "oral diagnosis" or "oral examination" or "oral health assessment" or "oral health care" or "oral health practice*" or "oral health promotion" or "oral health screening" or "oral health status").ti. | 13884 |
|  | #3 | (guideline* or guidance* or consensus* or recommendation* or "best practice*" or statement* or standard* or "practice parameter*" or "position paper*" or "position stand*" or "position statement*").ti. | 339577 |
|  | #4 | #1 AND #2 AND #3 | 10 |
|  | #5 | #4 Filters: Date: 1/1/2010 – 31/12/2020, English. | 10 |
| **Scopus** | #1 | ( TITLE-ABS-KEY-AUTH ( "prenatal care" ) OR TITLE-ABS-KEY ( "antenatal care" ) OR TITLE-ABS-KEY ( "perinatal care" ) OR TITLE-ABS-KEY ( prenatal ) OR TITLE-ABS-KEY ( antenatal ) OR TITLE-ABS-KEY ( perinatal ) OR TITLE-ABS-KEY ( maternal ) OR TITLE-ABS-KEY ( pregnanc* ) OR TITLE-ABS-KEY ( obstetric ) ) | 1355132 |
|  | #2 | ( TITLE-ABS-KEY-AUTH ( "oral health" ) OR TITLE-ABS-KEY ( "dental assessment" ) OR TITLE-ABS-KEY ( "oral disease*" ) OR TITLE-ABS-KEY ( "oral diagnosis" ) OR TITLE-ABS-KEY ( "oral examination" ) OR TITLE-ABS-KEY ( "oral health assessment" ) OR TITLE-ABS-KEY ( "oral health care" ) OR TITLE-ABS-KEY ( "oral health counsel*" ) OR TITLE-ABS-KEY ( "oral health practice*" ) OR TITLE-ABS-KEY ( "oral health promotion" ) OR TITLE-ABS-KEY ( "oral health status" ) OR TITLE-ABS-KEY ( "oral health screen*" ) ) | 44369 |
|  | #3 | ( TITLE-ABS-KEY-AUTH ( guideline* ) OR TITLE-ABS-KEY ( guidance* ) OR TITLE-ABS-KEY ( recommendation* ) OR TITLE-ABS-KEY ( consensus* ) OR TITLE-ABS-KEY ( standard* ) OR TITLE-ABS-KEY ( statement* ) OR TITLE-ABS-KEY ( "best practice*" ) OR TITLE-ABS-KEY ( "practice parameter*" ) OR TITLE-ABS-KEY ( "position paper*" ) OR TITLE-ABS-KEY ( "position stand*" ) OR TITLE-ABS-KEY ( "position statement*" ) ) | 5640307 |
|  | #4 | #1 AND #2 AND #3 | 277 |
|  | #5 | #4 Filters: Date: 1/1/2010 – 31/12/2020, English. | 189 |
| **Database Total** |  |  | 231 |
| After duplicates removed in EndNote X9.3.1 |  |  | 57 |
| **Total** |  | Electronic databases | 57 |

GREY LITERATURE SEARCH STRATEGIES

Records were searched and reviewed within Google Search engine, professional organisation website, or guideline database. Relevant records were obtained for further review to check for eligibility for inclusion. A secondary Google search was undertaken in 20/04/2021 to check for further/updated relevant records.

| **Table S2A.** Initial Google search strategy and results – 16/10/2020. | | | |
| --- | --- | --- | --- |
| **Database** | **Step** | **Search strategy & keywords** | **Results**  **(16/10/2020)** |
| **Grey Literature**  **Google search engine** | #1 | antenatal oral health guidelines | 146000 |
|  | #2 | perinatal oral health guidelines | 371000 |
|  | #3 | prenatal oral health guidelines | 303000 |
|  | #4 | pregnancy oral health guidelines | 48700000 |
|  | #5 | maternal oral health guidelines | 28200000 |
|  |  | #1-#5 Filters: Custom date range: 01/01/2010 – 31/12/2020, first 15 pages (10 per page) of each search by relevancy. | 750 |

| **Table S3A.** Secondary Google search strategy and results – 20/04/2021. | | | |
| --- | --- | --- | --- |
| **Database** | **Step** | **Search strategy & keywords** | **Results**  **(20/04/2021)** |
| **Grey Literature**  **Google search engine** | #1 | antenatal oral health guidelines | 147000 |
|  | #2 | perinatal oral health guidelines | 3290000 |
|  | #3 | prenatal oral health guidelines | 318000 |
|  | #4 | pregnancy oral health guidelines | 53100000 |
|  | #5 | maternal oral health guidelines | 32200000 |
|  |  | #1-#5 Filters: Date: 1/1/2010 – 20/04/2021, first 15 pages (10 per page) of each search by relevancy. | 750 |

| **Table S4A.** Purposive search strategy in relevant professional and guideline databases. | | | | |
| --- | --- | --- | --- | --- |
| **Professional and guideline development groups databases** | **Country /region** | **Step** | **Search strategy & keywords** | **Results**  **(16/10/2020 – 23/10/2020)** |
| Australian Government Department of Health | Australia | #1 | pregnancy + oral health + guideline | 550 |
|  |  | #2 | #1 Filters: first 2 pages (10 per page) by relevancy | 20 |
| Department of Health - Victoria | Australia | #1 | pregnancy + oral health + guideline | 830 |
|  |  | #2 | #1 Filters: first 2 pages (10 per page) by relevancy | 20 |
| Department of Health – Australian Capital Territory | Australia | #1 | pregnancy + oral health + guideline | 3 |
|  |  | #2 | #1 Filters: #1 Filters: first 2 pages (10 per page) by relevancy | 3 |
| Department of Health – Tasmania | Australia | #1 | pregnancy + oral health + guideline | 540 |
|  |  | #2 | #1 Filters: first 2 pages (10 per page) by relevancy | 20 |
| Department of Health – New South Wales | Australia | #1 | pregnancy + oral health + guideline | 7982 |
|  |  | #2 | #1 Filters: first 2 pages (10 per page) by relevancy | 20 |
| Department of Health – Northern Territory | Australia | #1 | pregnancy + oral health + guideline | 36 |
|  |  | #2 | #1 Filters: first 2 pages (10 per page) by relevancy | 20 |
| Department of Health – South Australia | Australia | #1 | pregnancy + oral health + guideline | 198 |
|  |  | #2 | #1 Filters: first 2 pages (10 per page) by relevancy | 20 |
| Department of Health – Queensland | Australia | #1 | pregnancy + oral health + guideline | 3114 |
|  |  | #2 | #1 Filters: first 2 pages (10 per page) by relevancy | 20 |
| Department of Health – Western Australia | Australia | #1 | pregnancy + oral health + guideline | 50 |
|  |  | #2 | #1 Filters: first 2 pages (10 per page) by relevancy | 20 |
| Australian National Health and Medical Research Council (NHMRC) | Australia | #1 | pregnancy + oral health + guideline | 41 |
|  |  | #2 | #1 Filters: first 2 pages (10 per page) by relevancy | 20 |
| Royal Australian College of General Practitioners (RACGP) | Australia | #1 | pregnancy + oral health + guideline | 2480 |
|  |  | #2 | #1 Filters: first 2 pages (10 per page) by relevancy | 20 |
| Royal Australian and New Zealand College of Obstetricians and Gynaecologists (RANZCOG) | Australia | #1 | pregnancy + oral health + guideline | 0 |
| New Zealand Guidelines Group | New Zealand | #1 | pregnancy + oral health + guideline | 181 |
|  |  | #2 | #1 Filters: first 2 pages (10 per page) by relevancy | 20 |
| National Institute of Clinical Excellence (NICE) | United Kingdom | #1 | pregnancy + oral health + guideline | 120 |
|  |  | #2 | #1 Filters: first 2 pages (10 per page) by relevancy | 20 |
| Royal College of Obstetricians and Gynaecologists (RCOG) | United Kingdom | #1 | pregnancy + oral health + guideline | 3 |
|  |  | #2 | #1 Filters: #1 Filters: first 2 pages (10 per page) by relevancy | 3 |
| Royal College of Midwives | United Kingdom | #1 | pregnancy + oral health + guideline | 0 |
| Royal College of Physicians | United Kingdom | #1 | pregnancy + oral health + guideline | 0 |
| The Scottish Intercollegiate Guidelines Network (SIGN) | United Kingdom | #1 | pregnancy + oral health + guideline | 36 |
|  |  | #2 | #1 Filters: first 2 pages (10 per page) by relevancy | 20 |
| American College of Obstetricians and Gynecologists (ACOG) | United States | #1 | pregnancy + oral health + guideline | 71 |
|  |  | #2 | #1 Filters: first 2 pages (10 per page) by relevancy | 20 |
| American College of Nurse-Midwives (ACNM) | United States | #1 | pregnancy + oral health + guideline | 10 |
|  |  | #2 | #1 Filters: #1 Filters: first 2 pages (10 per page) by relevancy | 10 |
| American College of Family Physicians (ACFP) | United States | #1 | pregnancy + oral health + guideline | 845 |
|  |  | #2 | #1 Filters: first 2 pages (10 per page) by relevancy | 20 |
| National Guideline Clearinghouse (NGC) | United States | #1 | pregnancy + oral health + guideline | 374 |
|  |  | #2 | #1 Filters: first 2 pages (10 per page) by relevancy | 20 |
| Society of Obstetricians and Gynaecologists of Canada (SOGC) | Canada | #1 | pregnancy + oral health + guideline | 107 |
|  |  | #2 | #1 Filters: first 2 pages (10 per page) by relevancy | 20 |
| Registered Nurses Association of Ontario (RNAO) | Canada | #1 | pregnancy + oral health + guideline | 309 |
|  |  | #2 | #1 Filters: first 2 pages (10 per page) by relevancy | 20 |
| World Health Organisation (WHO) | International | #1 | pregnancy + oral health + guideline | 0 |
| Guidelines International Network (G-I-N) | International | #1 | pregnancy + oral health + guideline | 0 |
| **Total** |  |  |  | 316 |
