# Supplementary material for: Clinical practice guidelines and consensus statements for antenatal oral healthcare: An assessment of their methodological quality and content of recommendations
Source: PLoS One. 2022 Feb 3;17(2):e0263444. doi: 10.1371/journal.pone.0263444 (PMC8812839; doi:10.1371/journal.pone.0263444)
Supplement: S2 Appendix — (DOCX) [file pone.0263444.s002.docx]

**S2 APPENDIX**

**Recommendation extraction forms of included guidance documents**

**Table S1A.** Recommendation extraction form using Australian Government Department of Health guideline.

| **Guidance document information** |  | |
| --- | --- | --- |
| Title | Clinical Practice Guidelines: Pregnancy Care | |
| Development organisation | Australian Government Department of Health | |
| Publication year | Published 2012, updated 2021 | |
| Guidance document type | Evidence-based guideline | |
| Guidance document methodology | Developed in accordance with National Health and Medical Research Council guideline development process | |
| Quality of evidence and grading of recommendations | National Health and Medical Research Council system | |
| Funding | Australian Government Department of Health | |
| Literature search date | 11.2010 | |
| Search strategy | Systematic literature search | |
| Research questions and supporting evidence | 1. Are there any dental procedures or treatment that are unsafe in pregnancy? | Informed knowledge:  1 RCT,  1 pseudo-RCT,  2 retrospective cohort studies,  1 cross-sectional study |
|  | 1. Does periodontal disease confer any risks to pregnancy or to the neonate? | Informed knowledge:  1 systematic review of RCTs,  8 RCTs,  7 retrospective cohort studies,  9 case-control studies,  14 cross-sectional studies |
|  | 1. Does dental caries confer any risks in pregnancy or to the neonate? | Informed knowledge:  2 retrospective cohort studies,  2 cross-sectional studies |
|  | 1. What is the optimal timing of screening for oral health? | No studies found. |
|  | 1. What information/education and advice should clinicians provide for women? | Informed Recommendation:  1 RCT,  5 case series/cohort studies |
|  | 1. What are the additional considerations for Aboriginal and Torres Strait Islander women? | No studies found. |
| Number of references | 27 | |
| Recommendation | At the first antenatal visit, advise women to have oral health checks and treatment, if required as good oral health is important to a woman’s health and treatment can be safely provided during pregnancy. | |
| Strength of recommendation | B: Body of evidence can be trusted to guide practice in most situations. | |
| Practice summary | 1. Discuss oral health with women: Explain that pregnancy does not cause dental problems but may make them more likely. Advise women to have their oral health checked and to tell the dentist that they are pregnant. | |
|  | 1. Provide advice on oral health to women experiencing nausea and vomiting: Explain that vomiting exposes teeth to acid and give tips on how to reduce the impact. | |

Abbreviation: RCT: randomised controlled trial.

**Table S2A.** Recommendation extraction form using American Academy of Pediatric Dentistry guideline.

| Guidance document information |  | |
| --- | --- | --- |
| Title | **Perinatal and Infant Oral Health Care** | |
| Development organisation | **American Academy of Pediatric Dentistry** | |
| Publication year | Published 2009, updated 2016 | |
| Guidance document type | Expert consensus | |
| Guidance document methodology | Developed through expert consensus of relevant professionals | |
| Quality of evidence and grading of recommendations | None | |
| Funding | None | |
| Literature search date | Unclear | |
| Search strategy | Comprehensive literature search | |
| Research questions and supporting evidence | None provided | Expert opinion |
| Number of references | 61 | |
| Recommendations | 1. Educate women regarding: | |
|  | - 1. Diet including the adequate quality and quantity of nutrients for the mother-to-be and the child. This education also should include information regarding the caries process and food cravings that may increase the mother’s caries risk. | |
|  | - 1. Comprehensive oral examination, dental prophylaxis, and treatment during pregnancy. Dental treatment during pregnancy, including dental radiographs with proper shielding and local anesthetic, is safe in all trimesters and optimal in the second trimester. Due to possible patient discomfort, elective treatment sometimes may be deferred until after delivery. | |
|  | - 1. Proper oral hygiene, using a fluoridated toothpaste, chewing sugar-free gum, and eating small amounts of nutritious food throughout the day to help minimize their caries risk. | |
|  | 1. Parents should be encouraged establish a dental home for infants by 12 months of age. | |
| Strength of recommendation | None | |
| Practice summary | None | |

**Table S3A.** Recommendation extraction form using California Dental Association Foundation guideline.

| Guidance document information |  | |
| --- | --- | --- |
| Title | **Oral Health During Pregnancy and Early Childhood: Evidence-based Guidelines for Health Professionals** | |
| Development organisation | **California Dental Association Foundation** | |
| Publication year | Published 2010 | |
| Guidance document type | Expert consensus | |
| Guidance document methodology | Developed through expert consensus of relevant professionals | |
| Quality of evidence and grading of recommendations | None | |
| Funding | California HealthCare Foundation, First 5 California Sierra Health Foundation, Anthem Blue Cross Foundation | |
| Literature search date | Unclear | |
| Search strategy | Critical review of established guidelines | |
|  | Comprehensive literature search for supporting information | |
| Research questions and supporting evidence | None provided | Expert opinion, consensus conference |
| Number of references | 249 | |
| Recommendations | 1. Educate the pregnant woman about the importance of her oral health, not only for her overall health, but also for the oral health of her children. | |
|  | 1. Provide education and dental referrals for oral health care, understanding that such care may have relatively low priority for some women, particularly those challenged by financial worries, unemployment, housing, intimate partner violence, substance abuse or other life-stressors. | |
|  | 1. Ask the woman if she has any concerns/fears about getting dental care while pregnant. Based on her response, be ready to inform her that dental care is safe during pregnancy and address specific concerns. | |
|  | 1. Advise the pregnant woman that: | |
|  | - 1. Prevention, diagnosis and treatment of oral diseases (including needed dental X-rays and use of local anesthesia) are highly beneficial and can be undertaken any time during pregnancy with no additional fetal or maternal risk as compared to not providing care. | |
|  | - 1. Dental care can improve her overall health and the health of her developing fetus and her children. | |
|  | 1. Determine and document in the prenatal record whether the patient is already under the care of an oral health professional; if a referral is needed, make a referral and document this in the prenatal record. | |
|  | 1. Encourage all women at the first prenatal visit to schedule a dental examination if one has not been performed in the past six months, or if a new condition has developed or is suspected. | |
|  | 1. Facilitate dental care by providing written consultation or an oral health referral form. While many medical providers understand there is no need for dentists to consult with an MD for routine dental care on a healthy patient, such a form from the obstetrical provider reassures the patient as well as the dentist that dental care is acceptable/permissible during pregnancy. Include this form as part of routine new-prenatal patient paperwork. | |
|  | 1. Obtain or develop and maintain a list of community dental referral sources that will provide services for pregnant women, particularly for women enrolled in publicly funded programs (e.g., Medicaid). | |
|  | 1. As a routine part of the initial prenatal examination, conduct and document an oral health assessment of the teeth, gums, tongue, palate and mucosa. | |
|  | 1. Share appropriate clinical information with the oral health professional and answer questions that the oral health professional may ask about a patient or condition. | |
|  | 1. Encourage and support all women to adhere to the oral health professional’s recommendations for appropriate treatment and follow-up care for oral disease. | |
|  | 1. Encourage and support a woman’s decision to breastfeed, providing appropriate oral hygiene instructions for after feeding, and have ready access to resources. | |
|  | 1. Educate women and encourage behaviors that support good oral health: | |
|  | - 1. Brushing teeth twice daily with fluoridated toothpaste, especially before bedtime, and flossing daily. | |
|  | - 1. Taking prenatal vitamins, including folic acid to reduce the risk of birth defects such as cleft lip and palate, and eating foods high in protein, calcium, phosphorus and vitamins A, C and D. | |
|  | - 1. Chewing xylitol-containing gum or other xylitol-containing products, four to five times a day, after eating. | |
|  | - 1. Not delaying necessary dental treatment. | |
|  | - 1. Limiting foods containing fermentable carbohydrates—sugars (including fruit sugars), cookies, crackers, chips—to mealtimes only. Frequent between-meal consumption of these foods increases caries risk. | |
|  | - 1. Limiting drinking juice, soda, sports drinks or carbonated drinks (including diet soda) between meals. These drinks contain sugar that can cause caries. Even diet sodas contain acids that can weaken the enamel of teeth, especially those containing caffeine and citric acid. | |
|  | 1. Advise pregnant women experiencing frequent nausea and vomiting to reduce erosion of tooth surfaces by: | |
|  | - 1. Eating small amounts of nutritious yet noncariogenic foods—snacks rich in protein, such as cheese—throughout the day. | |
|  | - 1. Using a teaspoon of baking soda (sodium bicarbonate) in a cup of water to rinse and spit after vomiting, avoiding tooth brushing directly after vomiting as the effect of erosion can be exacerbated by brushing an already demineralized tooth surface. | |
|  | - 1. Using gentle tooth brushing and fluoride toothpaste twice daily to prevent damage to demineralized tooth surfaces. | |
|  | - 1. Using a fluoride-containing mouth rinse immediately before bedtime to help remineralize teeth. | |
|  | 1. Advise women that the following actions may reduce the risk of caries in their children: | |
|  | - 1. Wiping an infant’s gums or teeth, especially along the gum line, with a soft cloth after breast or bottle feeding. | |
|  | - 1. Brushing the child’s teeth using a pea-sized (the size of a child’s pinky nail) amount of toothpaste, especially before bedtime. Children older than 2 should use fluoride toothpaste; children younger than 2 should use a smear of fluoride toothpaste on the brush only if they are at moderate to high risk of developing caries. | |
|  | - 1. Helping a child brush their teeth until they are about 7 years old. | |
|  | - 1. Avoiding putting the infant to bed with a bottle or sippy cup containing anything other than water. | |
|  | - 1. Avoiding saliva-sharing behaviors, such as kissing the baby on the mouth, sharing a spoon when tasting baby food, cleaning a dropped pacifier by mouth or wiping the baby’s mouth with a cloth moistened with saliva. For older children, avoiding the sharing of straws, cups or utensils. | |
|  | - 1. Using a bottle or sippy cup between meals containing only water. | |
|  | - 1. Begin weaning children from at-will bottle and sippy cup use (such as in an effort to pacify a child’s behavior) by about 12 months of age. | |
|  | - 1. Choosing fresh fruit rather than fruit juice to meet the recommended daily fruit intake. | |
|  | - 1. Regularly lifting the lip and looking in their child’s mouth for white or brown spots on the teeth. | |
|  | 1. Encourage women to learn more about oral health during pregnancy and early childhood by accessing available consumer information including reputable web sites. | |
|  | 1. Advise and encourage the woman to obtain necessary follow-up dental care and oral health maintenance during the postpartum period and thereafter. | |
| Strength of recommendation | None | |
| Practice summary | None | |

**Table S4A.** Recommendation extraction form using European Federation of Periodontology guideline.

| Guidance document information |  | |
| --- | --- | --- |
| Title | **The Relationship Between Oral Health and Pregnancy: Guidelines for Non-dentistry Health Professionals** | |
| Development organisation | **European Federation of Periodontology** | |
| Publication year | Published 2020 | |
| Guidance document type | Expert consensus | |
| Guidance document methodology | Developed through expert consensus of relevant professionals | |
| Quality of evidence and grading of recommendations | None | |
| Funding | Oral-B | |
| Literature search date | Unclear | |
| Search strategy | Comprehensive literature search for supporting information | |
| Research questions and supporting evidence | None provided | Expert opinion |
| Number of guidelines | None clearly provided within guideline. | |
| Recommendations | 1. Oral-health education: As part of their regular care, health professionals should provide oral-health education and oral-health screening to pregnant women. | |
|  | 1. Oral-health history: Health professionals should include an oral-health history as part of the patient’s general health history, including questions such as: Do you have swollen gums? Do you have problems eating or chewing food? Are you suffering from oral pain or other oral problems? | |
|  | 1. Oral-health examination: Health professionals should include an oral examination as part of their regular medical examination. This examination should assess the presence of bleeding in the margin between the teeth and the gingiva and overt gingival inflammation by asking the patient to open her mouth under a direct light and with the use of a tongue depressor. In the presence of these signs, the physician should refer the patient to an oral-health professional for adequate diagnosis and care. | |
|  | 1. Gingival inflammatory changes: Health professionals should inform women of the frequent gingival inflammatory changes associated with pregnancy and assure them that these changes are not physiological and that they should be appropriately diagnosed and treated by an oral-health professional. | |
|  | 1. Adverse pregnancy outcomes: Health professionals should also inform women that, in the presence of periodontitis, there may be a higher risk of adverse pregnancy outcomes. They should therefore always recommend that pregnant women visit an oral-health professional for a check-up early during gestation. This should be further emphasized to pregnant women at risk of adverse pregnancy outcomes because periodontal therapy may reduce the incidence of such outcomes. | |
|  | 1. Pre-pregnancy: Health professionals who treat women who want to become pregnant should also recommend that their patients visit an oral-health professional and establish healthy periodontal conditions before pregnancy, because this may favor the outcome of the planned pregnancy. In this regard, adverse pregnancy outcomes and periodontal disease share some important risk factors (such as smoking), so a healthy lifestyle should be encouraged and promoted. | |
| Strength of recommendation | None | |
| Practice summary | None | |

**Table S5A.** Recommendation extraction form using NACCHO/RACGP guideline.

| Guidance document information |  |
| --- | --- |
| Title | **National Guide to Preventive Health: Assessment for Aboriginal and Torres Strait Islander People** |
| Development organisation | **National Aboriginal Community Controlled Health Organisation/the Royal Australian College of General Practitioners (NACCHO/RACGP)** |
| Publication year | Published 2005, updated 2018 |
| Guidance document type | Evidence-based guideline |
| Guidance document methodology | Developed in accordance with National Health and Medical Research Council guideline development process |
| Quality of evidence and grading of recommendations | National Health and Medical Research Council system |
| Funding | None |
| Literature search date | 2016, 2017 |
| Search strategy | Critical review of established guidelines |
|  | Comprehensive literature search for supporting information |
| Research questions and supporting evidence | None specific to oral health. |
|  | *Clinical Practice Guidelines: Pregnancy Care* from the Australian Government Department of Health, version 2012 used to inform development of Recommendation. |
| Number of references | 13 |
| Recommendation | 1. At the first antenatal visit, advise women to have an oral health check and treatment if required. |
|  | 1. Assessment: |
|  | - 1. Visually inspect teeth for evidence of caries, periodontal disease, assessment of maternal caries and/or poor oral hygiene. |
|  | - 1. Assess oral hygiene practices and consumption of sucrose and sweetened drinks, especially in baby bottles, ‘honey on the dummy’ or other sweet substances such as glycerine on the dummy, and intake of sugared medicines. |
|  | - 1. Assess access to fluoridated water supply advice. |
|  | - 1. Brush teeth twice daily with a soft toothbrush and fluoride toothpaste and advise to spit, not rinse, excess paste. |
|  | - 1. Advise about the hazards of high carbohydrate and acidic snacks and drinks taken between meals. |
|  | - 1. Advise against high and regular consumption of black cola, sweetened fizzy drinks and sports drinks, with water being the preferred drink. |
|  | - 1. Promote breastfeeding, with weaning to a baby cup, not a bottle. |
|  | - 1. If bottles are used, advise against the use of any fluid apart from water and do not put baby to sleep with a bottle. |
|  | - 1. Advise about smoking cessation and limiting alcohol consumption. |
|  | - 1. Use sugar-free chewing gum for saliva stimulation. |
|  | - 1. Use a mouth guard when playing contact sport. |
|  | - 1. Recommend regular dental check-up. |
| Strength of recommendation | For recommendation 1: B: Body of evidence can be trusted to guide practice in most situations. |
| Practice summary | At the first antenatal visit, undertake an oral health review including the assessment of teeth, gums and oral mucosa, as part of a regular health check. |

**Table S6A.** Recommendation extraction form using OHCDPEW guideline.

| Guidance document information |  | |
| --- | --- | --- |
| Title | **Oral Health During pregnancy: A National Consensus Statement** | |
| Development organisation | **Oral Health Care During Pregnancy Expert Workgroup (OHCDPEW)** | |
| Publication year | Published 2012 | |
| Guidance document type | Expert consensus | |
| Guidance document methodology | Developed through expert consensus of relevant professionals | |
| Quality of evidence and grading of recommendations | None | |
| Funding | Maternal and Child Health Bureau, Health Resources and Services Administration, United States Department of Health and Human Services | |
| Literature search date | Unclear | |
| Search strategy | Critical review of established guidelines | |
|  | Comprehensive literature search for supporting information | |
| Research questions and supporting evidence | None provided | Expert opinion, consensus conference |
| Number of references | None clearly provided within guideline. | |
| Recommendations | 1. Assess pregnant women’s oral health status: During the initial prenatal evaluation: | |
|  | - 1. Take an oral health history. Following are examples of questions that prenatal care health professionals may ask pregnant women. | |
|  | - 1. Check the mouth for problems such as swollen or bleeding gums, untreated dental decay (tooth with a cavity), mucosal lesions, signs of infection (e.g., a draining fistula), or trauma. | |
|  | - 1. Document your findings in the woman’s medical record. | |
|  | 1. Advise pregnant women about oral health care: | |
|  | - 1. Reassure women that oral health care, including use of radiographs, pain medication, and local anesthesia, is safe throughout pregnancy. | |
|  | - 1. If the last dental visit took place more than 6 months ago or if any oral health problems were identified during the assessment, advise women to schedule an appointment with a dentist as soon as possible. If urgent care is needed, write and facilitate a formal referral to a dentist who maintains a collaborative relationship with the prenatal care health professional. | |
|  | - 1. Encourage women to seek oral health care, practice good oral hygiene, eat healthy foods, and attend prenatal classes during pregnancy. | |
|  | - 1. Counsel women to follow oral health professionals’ recommendations for achieving and maintaining optimal oral health. | |
|  | 1. Work in collaboration with oral health professionals: | |
|  | - 1. Establish relationships with oral health professionals in the community. Develop a formal referral process whereby the oral health professional agrees to see the referred individual in a timely manner (e.g., that day, the following day) and to provide subsequent care. | |
|  | - 1. Share pertinent information about pregnant women with oral health professionals, and coordinate care with oral health professionals as appropriate. | |
|  | 1. Provide support services (case management) to pregnant women: | |
|  | - 1. Help pregnant women complete applications for insurance or other sources of coverage, social services (e.g., domestic violence services), or other needs (e.g., transportation, translation). | |
|  | - 1. If the woman does not have a dental home, explain the importance of optimal oral health during pregnancy. Help her obtain care by facilitating referrals to oral health professionals in the community, including those who serve pregnant women enrolled in Medicaid and other public insurance programs, or by contacting a dental office to schedule care. | |
|  | 1. Improve health services in the community: | |
|  | 1. On the patient-intake form, include questions about oral health (e.g., name and contact information of oral health professional, reason for and date of last dental visit, previous dental procedures). | |
|  | 1. Establish partnerships with community-based programs that serve pregnant women with low incomes. | |
|  | 1. Provide a referral to a nutrition professional if counseling (e.g., guidance on food choices or nutrition-related health problems) would be beneficial. | |
|  | 1. Integrate oral health topics into prenatal classes. | |
|  | 1. Provide culturally and linguistically appropriate care. Take the time to ensure that women understand the information shared with them. | |
| Strength of recommendation | None | |
| Practice summary | None | |

**Table S7A.** Recommendation extraction form using Perinatal Services British Columbia guideline.

| Guidance document information |  | |
| --- | --- | --- |
| Title | **Provincial Perinatal Guidelines: Population and Public Health Prenatal Care Pathway** | |
| Development organisation | **Perinatal Services British Columbia** | |
| Publication year | Published 2014 | |
| Guidance document type | Expert consensus | |
| Guidance document methodology | Developed through expert consensus of relevant professionals | |
| Quality of evidence and grading of recommendations | None | |
| Funding | None | |
| Literature search date | Unclear | |
| Search strategy | Comprehensive literature search for supporting information | |
| Research questions and supporting evidence | None provided | Expert opinion |
| Number of references | 3 | |
| Recommendations | Assess: woman’s knowledge related to recommended oral health care during pregnancy and her ability to access dental health care. | |
|  | 1. Norm: Woman has knowledge of the importance of oral health during pregnancy, the capacity, skills and tools to support oral health, and has contact with, or plans to contact a dental care professional. | |
|  | 1. Client education/anticipatory guidance: Advise woman that: | |
|  | - 1. Oral health care is important for the prevention of tooth decay, periodontal disease and to prevent transmission of oral bacteria that may cause tooth decay for her child. She should brush with a fluoride toothpaste at least twice daily and floss daily. | |
|  | - 1. Dental care (including x-rays and local anaesthetic if needed) is safe for all pregnant women. | |
|  | - 1. Women experiencing vomiting in pregnancy (“morning sickness”) should avoid brushing for an hour after vomiting to protect tooth enamel but can rinse their mouths with water or fluoride mouth wash. | |
|  | 1. Variance: Woman lacks knowledge of the importance of oral health and its potential impact on pregnancy; Woman lacks the capacity, skills or tools to perform personal oral care; Woman has barriers (e.g. financial, low literacy, language or transportation etc.) prohibiting her access to dental care. | |
|  | 1. Intervention: | |
|  | - 1. Screening and referral: Screen woman for concerns related to oral health and access to oral health care; refer to local dental health professionals as indicated. Note: It is particularly important to assess a woman with chronic health conditions such as diabetes or heart disease for oral health. | |
|  | - 1. Health education: Offer woman information about the importance of oral health in pregnancy and about how and where she can access dental health services. | |
|  | - 1. Capacity building: Support and assist a vulnerable woman and those needing help due to barriers or lack of skills to address oral health concerns, including referral to dental health providers and supporting her to access care. Support woman to build knowledge and capacity to manage life-long oral health promoting habits for herself and her family. | |
|  | - 1. Care coordination: Communicate and collaborate with the local resources to facilitate access to dental care for women with barriers. | |
| Strength of recommendation | None | |
| Practice summary | Assess: woman’s knowledge related to recommended oral health care during pregnancy and her ability to access dental health care. | |
