# Supplementary material for: Clinical practice guidelines and consensus statements for antenatal oral healthcare: An assessment of their methodological quality and content of recommendations
Source: PLoS One. 2022 Feb 3;17(2):e0263444. doi: 10.1371/journal.pone.0263444 (PMC8812839; doi:10.1371/journal.pone.0263444)
Supplement: S1 Table — As of 10.2020. (DOCX) [file pone.0263444.s003.docx]

| **List** | **Country** |
| --- | --- |
| 1 | Australia |
| 2 | Austria |
| 3 | Belgium |
| 4 | Canada |
| 5 | Chile |
| 6 | Colombia |
| 7 | Czech Republic |
| 8 | Denmark |
| 9 | Estonia |
| 10 | Finland |
| 11 | France |
| 12 | Germany |
| 13 | Greece |
| 14 | Hungary |
| 15 | Iceland |
| 16 | Ireland |
| 17 | Israel |
| 18 | Italy |
| 19 | Japan |
| 20 | Korea |
| 21 | Latvia |
| 22 | Lithuania |
| 23 | Luxembourg |
| 24 | Mexico |
| 25 | Netherlands |
| 26 | New Zealand |
| 27 | Norway |
| 28 | Poland |
| 29 | Portugal |
| 30 | Slovak Republic |
| 31 | Slovenia |
| 32 | Spain |
| 33 | Sweden |
| 34 | Switzerland |
| 35 | Turkey |
| 36 | United Kingdom |
| 37 | United States |
