# Supplementary material for: Clinical practice guidelines and consensus statements for antenatal oral healthcare: An assessment of their methodological quality and content of recommendations
Source: PLoS One. 2022 Feb 3;17(2):e0263444. doi: 10.1371/journal.pone.0263444 (PMC8812839; doi:10.1371/journal.pone.0263444)
Supplement: S2 Table — (DOCX) [file pone.0263444.s004.docx]

| **S2 Table.** Grading of recommendations in evidence-based guidelines (n=2). | | | | | | | |
| --- | --- | --- | --- | --- | --- | --- | --- |
| **Development organisation** | **Recommendation** | **Body of evidence matrix^*^** | | | | | **Grade of recommendation^*^** |
|  |  | Evidence base | Consistency | Clinical impact | Generalisability | Applicability |  |
| Australian Government Department of Health [22] | At the first antenatal visit, advise women to have oral health checks and treatment, if required as good oral health is important to a woman’s health and treatment and can be safely provided during pregnancy | **A:** several randomised controlled trials with low risk of bias, with one or more systematic reviews and meta-analysis of interventional studies with low risk of bias | **B:** most studies demonstrate consistency of results | **C:** substantial benefit to target population | **B:** studied population in the body of evidence are similar to target guideline population | **A:** directly applicable to Australian healthcare context | **B:** body of evidence can be trusted to guide practice in most situations |
| National Guide to Preventive Health: Assessment for Aboriginal and Torres Strait Islander People [46] | At the first antenatal visit, advise women to have an oral health check and treatment if required | **A:** several interventional studies with low risk of bias, with one or more systematic reviews and meta-analyses of interventional studies with low risk of bias | **B:** most studies demonstrate consistency of results | **C:** substantial benefit to target population | **B:** studied population in the body of evidence are similar to target guideline population | **A:** directly applicable to Australian healthcare context | **B:** body of evidence can be trusted to guide practice in most situations |

* Based on National Health and Medical Research Council system.
