# Supplementary material for: Clinical practice guidelines and consensus statements for antenatal oral healthcare: An assessment of their methodological quality and content of recommendations
Source: PLoS One. 2022 Feb 3;17(2):e0263444. doi: 10.1371/journal.pone.0263444 (PMC8812839; doi:10.1371/journal.pone.0263444)
Supplement: S1 File — (DOCX) [file pone.0263444.s006.docx]

**LIST OF PROFESSIONAL SOCIETY WEBSITES**

American Academy of Pediatric Dentistry: <www.aapd.org>

American College of Obstetricians and Gynecologists: <www.acog.org>

Australian Government Department of Health: <www.health.gov.au>

California Dental Association: <www.cda.org>

European Federation of Periodontology: <www.efp.org>

National Aboriginal Community Controlled Health Organisation: <www.naccho.org.au>

National Maternal and Child Oral Health Resource Centre: <www.mchoralhealth.org>

Perinatal Services British Columbia: <www.perinatalservicesbc.ca>

Royal Australian College of General Practitioners: <www.racgp.org.au>
